# Analysis of pressure flotation mechanisms and their practical application in the treatment of metal-containing wastewater

**DOI:** 10.1038/s41598-026-39418-2

**Published:** 2026-02-13

**Authors:** Viktor Fylypchuk, Galyna Kalda, Volodymyr Anopolskyi, Leonid Fylypchuk, Małgorzata Kida, Ahmad Ali AlZubi

**Affiliations:** 1 Department of Occupational Health and Safety, National University of Water and Environmental Engineering, 11, Soborna St. 33028, Rivne, Ukraine; 2 Department of Water Supply and Sewage Systems, Faculty of Civil and Environmental Engineering and Architecture, Rzeszow University of Technology, Ave Powstańców Warszawy 12, Rzeszów, 35-959 Poland; 3 Department of Construction and Civil Security, Khmelnitskiy National University, 11, Instytutska street, Khmelnitskiy, 29016 Ukraine; 4 Research Engineering Company «Elf», 31, Vidinskaya St., 33018, Rivne, Ukraine; 5 Department of Energy, Institute of Energy, Automation and Water Management, National University of Water and Environmental Engineering, 11, Soborna St., Rivne, Ukraine; 6 Department of Chemistry and Environmental Engineering, Faculty of Civil and Environmental Engineering and Architecture, Rzeszow University of Technology, Ave Powstańców Warszawy 12, Rzeszów, 35-959 Poland; 7 Department of Computer Science and Engineering, College of Applied Sciences, King Saud University, Riyadh, Saudi Arabia

**Keywords:** Dissolved air flotation (DAF), Metal-containing water, Metal hydroxides, Selective purification, Multichamber flotation units, Chemistry, Engineering, Environmental sciences, Hydrology

## Abstract

This article examines the fundamental patterns of the pressure flotation process by analyzing the relationships identified by the authors. These relationships link the gas content in the flotation unit with bubble size and the size of floating particles, as well as their respective concentrations and densities. On the basis of this analysis, the optimal conditions for applying pressure flotation to the treatment of metal-containing water are determined. The use of experimentally determined gas‒solid (*G/S*) ratios to calculate key parameters of the pressure flotation process for treating wastewater containing various metals is validated. The main innovation of this work lies in the development of multichamber flotation units designed to optimize flow dynamics and maximize particle-bubble collision efficiency. By incorporating sequential zonal separation and tailored turbulence control, these units enable the selective removal of metals from wastewater and the production of metal-rich sludge suitable for safe disposal. The practical application of selective pressure flotation for the treatment of metal-containing water, particularly at medium and high metal concentrations, is highly promising.

## Introduction

Organic and inorganic contaminants are increasingly being identified in aquatic ecosystems, posing a significant threat to the environmental functioning and health of aquatic organisms. These substances may lead to long-term disturbances in the ecological balance. As reported in the literature^1^, waters originating from urbanized areas, particularly mixtures of domestic wastewater and stormwater runoff, contain numerous organic compounds classified as micropollutants. One of the major sources of such contamination is the release of plastic materials into the aquatic environment. Studies presented in^2^have demonstrated that waste originating from linear sewer systems constitutes a significant source of plasticizer pollution in aquatic environments, highlighting the role of sewer infrastructure as an important transport pathway for these substances.

Pressure flotation is one of the most effective phase separation methods used in wastewater treatment for the removal of various pollutants. Pressure flotation systems have been developed and successfully applied for the treatment of wastewater generated by oil refineries, machine-building plants, light industry, the food industry, and other industrial sectors. In practice, proprietary flotation units manufactured from steel or plastic are most commonly employed. These units are characterized by operational simplicity, low maintenance requirements, and a high degree of automation, with sludge removal achieved through collection mechanisms of various designs.

Experience has also been gained in the application of pressure flotation for the treatment of metal-containing wastewater generated by electroplating facilities and printed circuit board manufacturing in the radio engineering, electronics, and instrumentation industries, among others^3,4^. This issue is particularly important, as many metals exhibit toxic, carcinogenic, mutagenic, and other adverse effects on humans, living organisms, and the environment.

The use of pressure flotation for the removal of dissolved metals after their conversion into poorly soluble compounds, primarily hydroxides, represents a highly relevant and promising approach in wastewater treatment technology. This process offers significant advantages over conventional settling and clarification in a suspended sediment layer, which are widely applied in global practice.

Compared with sediment formed during settling, metal-containing sludge generated by flotation is characterized by a lower moisture content and smaller volume. For example, the moisture content of flotation sludge typically ranges from 88 to 92%, whereas that of settled sediment is 96–98%. As a result, the volume of sludge produced after flotation treatment is 3–6 times lower than that of sediment, which significantly reduces the environmental burden associated with the storage of toxic substances. Moreover, owing to the presence of gas bubbles, the specific filtration resistance of flotation sludge is considerably lower than that of settled sediment, and no additional chemical conditioning is required prior to dewatering. Consequently, the productivity of dewatering equipment is 1.5–2 times greater for flotation sludge than for sediment.

The average settling time ranges from 1.5 to 3.0 h, whereas the flotation process typically requires only 20–30 min. This substantial reduction in treatment time leads to smaller equipment volumes, lower capital costs, and reduced land requirements for wastewater treatment facilities. Pressure flotation is particularly effective for wastewater containing medium to high concentrations of metals, as during settling processes, a significant fraction of metal hydroxide flocs may be entrained and discharged with the treated effluent.

An additional advantage of pressure flotation, as demonstrated by experimental studies, is the significantly lower degree of metal ion leaching, such as copper(II), iron(III), chromium(III), and nickel(II), from flotation sludge than from sediment obtained by reagent precipitation^5^. This property enables long-term storage of flotation sludge in industrial waste landfills or its disposal with minimal risk of metal release into the environment.

Pressure flotation is also an environmentally friendly treatment process, as it does not involve the use of harmful gases, and the treated water becomes saturated with dissolved oxygen, which inhibits the development of anaerobic processes.

In summary, pressure flotation offers substantial environmental and economic advantages over reagent settling. Overall, it meets the key criteria for achieving environmental protection objectives, provided that its technical feasibility is confirmed, in accordance with the European Union’s Best Available Techniques (BAT) framework^6^.

At present, a wide range of pressure flotation systems incorporating various technological and engineering solutions have been proposed^7–11^. An analysis of the available approaches indicates that single- or multistage circulating reagent-saturation pressure flotation systems are the most effective for the treatment of metal-containing water. In multistage reagent systems, fractional alkali dosing can be applied to enable stepwise flotation of poorly soluble metal compounds formed at different pH levels in aqueous media^12^. In addition, a technology has been developed for the treatment of high-temperature metal-containing wastewater, which involves cooling recirculating water to increase its dissolved air saturation under elevated pressure.

Flotation units are traditionally designed on the basis of either the flotation time, which is typically assumed to range from 15 to 40 min, or the hydraulic loading rate, which is usually within 3–10 m³/(m²·h). The specific values of these design parameters depend on the concentration of impurities in the treated water and the amount of dissolved air in the recirculation stream downstream of the saturator. Therefore, the gas-to-solid (*G/S*) ratio, defined as the ratio of the mass of dissolved air to the mass of particles entering the flotation unit per unit time, is commonly used as an additional design parameter. Experimental studies reported in^13^demonstrated that the maximum purification efficiency is achieved at *G/S* values in the range of 0.02–0.06. For design calculations, however, a broader range of *G/S* values (0.005–0.060) may be applied. Nevertheless, data describing the dependence of the *G/S* ratio on the size and density of floating particles are lacking. In existing mathematical models of the pressure flotation process^14^, the particle size and density are considered only as parameters influencing the flotation velocity of aggregate particles.

Effective treatment of metal-containing water by pressure flotation can be achieved only through the application of specific technological and technical measures. The degree of metal removal strongly depends on the concentration of metals in wastewater, the completeness of the formation of poorly soluble metal compounds following alkalization, and the gas content in the flotation unit^15^.

However, the influence of gas content on flotation efficiency, as a function of the properties of poorly soluble metal compounds formed during alkalization, has not yet been sufficiently investigated from a theoretical perspective. This knowledge gap is particularly evident with respect to the key characteristics of these compounds, such as their particle size and density.

This study presents a novel approach to investigate and analyze the theoretical principles governing the pressure flotation process in a multi-chamber configuration. In particular, it introduces a new framework for elucidating the influence of gas content, as well as the concentration, size, and density of poorly soluble metal compounds, on process performance. The novelty of this work lies in the development of a multi-chamber flotation unit of original design, which, unlike traditional single-stage systems, incorporates innovative technical solutions for selective metal extraction and significantly improved energy efficiency.

## Results and discussion

### Characteristics and gas solubility in pressure flotation

One characteristic feature of pressure flotation is the extremely short period of bubble formation in an air-saturated liquid when the pressure is reduced from elevated to atmospheric values. To ensure a more uniform distribution of bubbles within the wastewater, rapid mixing with the recirculated water is therefore needed. Under such conditions, some bubble nuclei may form directly on the hydrophobic surfaces of the particles being floated.

The total number of bubbles generated in the flotation unit after mixing the recirculated water with the wastewater depends on the concentration of gases dissolved in the recirculation stream, which constitute air, as well as on the velocity at which the air-saturated water exits the throttling device and the resulting bubble size distribution. The main components of air are nitrogen (78.09% by volume or 75.50% by mass) and oxygen (20.95% by volume or 23.10% by mass)^16^.

The maximum concentrations of nitrogen and oxygen dissolved in the recirculation water can be determined via Henry’s law^17^.

1 $$\:{C}_{g\left(i\right)}={K}_{g\left(i\right)}{P}_{g\left(i\right)},\mathrm{m}\mathrm{o}\mathrm{l}/\mathrm{d}\mathrm{m}^3$$

where $\:{C}_{g\left(i\right)}$ is the concentration of the i-th air component in saturated recirculating water, mol/l; $\:{K}_{g\left(i\right)}$ is Henry’s constant for the *i*-th air component, mol/(dm^3^·Pa); and $\:{K}_{g\left(i\right)}$ is the pressure of the *i*-th air component above the surface of the recirculation water, Pa.

On the basis of Eq. (1), the volume concentration of each dissolved air component, $\:{W}_{g\left(i\right)}$ (Eq. (2)), in the recirculation water can be determined. For this purpose, instead of Henry’s constant, which expresses the molar solubility of individual substances, it is more appropriate to use the specific volumetric solubility of air or its individual components in water $\:{\omega\:}_{g\left(i\right)}$.

2 $$\:{W}_{g\left(i\right)}={\omega\:}_{g\left(i\right)}{P}_{g\left(i\right)}/\mathrm{0,101335},\mathrm{c}\mathrm{m}^3/\mathrm{d}\mathrm{m}^3\:(\mathrm{d}\mathrm{m}^3/\mathrm{m}^3)$$

where $\:{\omega\:}_{g\left(i\right)}$- is the specific solubility of the i-th component of air, cm³/dm³ (dm³/m³), at a gas temperature of 0 °C and a pressure of 101,335 Pa (0.101335 MPa), depending on the water temperature (Table 1);$\:\:{P}_{g\left(i\right)}$ - is the partial pressure of the *i*-th air component above the surface of the recirculation water, Pa (MPa).

**Table 1:** Solubility of nitrogen and oxygen at a temperature of 0°С and a pressure of 0.101335 MPa, cm^3^/dm^3^ (dm^3^/m^3^) depending on the water temperature^18^.

Gas	Water temperature, °C								
	0	10	20	30	40	50	60	80	100
Nitrogen – N_2_	23,3	18,3	15,1	12,8	11,0	9,6	8,2	5,1	0
Oxygen – О_2_	48,9	38,0	31,0	26,1	23,1	20,9	19,5	17,6	17,0

When dissolved in water, nitrogen and oxygen saturate proportionally to their partial pressures, which can be determined via the following Eq. (3):

3 $$\:{P}_{g\left(i\right)}=\:{b}_{rg\left(i\right)}{P}_{air},\:\mathrm{M}\mathrm{P}\mathrm{a},$$

where $\:{b}_{rg\left(i\right)}$ is the relative volume content of the *i*-th component in the air and where $\:{P}_{air}-\:$ is the air pressure in megapascals (MPa).

Given that the volume fraction of nitrogen in air is 78.09% and that that of oxygen is 20.95%, their partial pressures under standard conditions can be calculated as follows:$\:\:{P}_{n}\:$= 0,7809 × 0,101335 = 0,079133 MPa, $\:{P}_{ox}\:$= 0,2095 × 0,101335 = 0,021230 MPa.

Taking these values into account, at a water temperature of 20 °C, the volume concentrations of the dissolved gases-nitrogen $\:{W}_{n}^{20}$, oxygen $\:{\:W}_{ox}^{20}$, and air $\:{W}_{air}^{20}$—under standard conditions can be calculated via Eq. (2), expressed in cm³/dm³ (or dm³/m³).

$$\:{W}_{n}^{20}={\omega\:}_{n}^{20}{P}_{n}/\mathrm{0,101335}=\mathrm{15,1}\cdot\mathrm{0,079133}/\mathrm{0,101335}\hspace{0.17em}=\hspace{0.17em}\mathrm{11,8}\:\mathrm{c}\mathrm{m}^3/\mathrm{d}\mathrm{m}^3\:(\mathrm{d}\mathrm{m}^3/\mathrm{m}^3)$$

$$\:{\:W}_{ox}^{20}={\omega\:}_{ox}^{20}{P}_{ox}/\mathrm{0,101335}=\mathrm{31,0}\cdot\mathrm{0,021230}/\mathrm{0,101335}\hspace{0.17em}=\hspace{0.17em}\mathrm{6,5}\:\mathrm{c}\mathrm{m}^3/\mathrm{d}\mathrm{m}^3\:(\mathrm{d}\mathrm{m}^3/\mathrm{m}^3)$$

4 $$\:{W}_{air}^{20}\:={W}_{n}^{20}+{W}_{ox}^{20}=\mathrm{11,8}\:+\:\mathrm{6,5}\hspace{0.17em}=\hspace{0.17em}\mathrm{18,3}\:\mathrm{c}\mathrm{m}^3/\mathrm{d}\mathrm{m}^3\:(\mathrm{d}\mathrm{m}^3/\mathrm{m}^3),\:$$

where $\:{\omega\:}_{n}^{20}$ and $\:{\omega\:}_{ox}^{20}$ are the solubilities of nitrogen and oxygen in water, respectively, under normal conditions and at a water temperature of 20°С (Table 1), cm^3^/dm^3^ (dm^3^/m^3^), and $\:{P}_{n},\:{P}_{ox}$ are the partial pressures of the nitrogen and oxygen contained in the air, respectively, in MPa.

Table 2 presents the calculated volume concentrations of nitrogen, oxygen, and air in water at a temperature of 0 °C and a pressure of 0.101335 MPa, which were determined via Eq. (2) and the data from Table 1. The calculations neglect the effects of air humidity and other minor components, whose total volume fraction is less than 1%.

**Table 2:** Estimated volume concentrations of nitrogen, oxygen, and air in water at 0 °C and an air pressure of 0.101335 MPa, expressed in cm³/dm³ (dm³/m³), as a function of water temperature.

Gas	Water temperature, °С								
	0	10	20	30	40	50	60	80	100
Nitrogen – N_2_	18,2	14,3	11,8	10,0	8,6	7,5	6,4	4,0	0
Oxygen – О_2_	10,2	8,0	6,5	5,5	4,8	4,4	4,1	3,7	3,6
Air	28,4	22,3	18,3	15,5	13,4	11,9	10,5	7,7	3,6

Previous studies have demonstrated that the presence and amount of dissolved gases directly affect flotation performance. Dissolved gases enhance the formation of bubble‒particle aggregates and reduce the induction time, leading to improved flotation efficiency. Moreover, physicochemical parameters such as temperature, pH, and solid concentration influence air dissolution and bubble size distribution, which are critical for flotation efficiency^19^.

Under real conditions, fluctuations in wastewater temperature can lead to changes in gas solubility. Additionally, the high concentrations of organic substances and divalent iron in wastewater result in partial consumption of dissolved oxygen due to oxidation reactions. Therefore, potential variations in nitrogen and oxygen concentrations during wastewater treatment should be taken into account in comparison with the calculated values presented in Table 2. Furthermore, when recirculating water is saturated with air in a saturator, factors such as the type of saturator, duration of air saturation, and temperature of the air must also be considered.

When recirculating water is saturated with air in a saturator, factors such as the type of saturator, duration of air saturation, and air temperature must be considered.

### Calculation of the gas content and bubble‒particle interactions

To analyze the behavior of the flotation process, it is necessary not only to determine the volume concentrations of air and its components (Table 2) but also to determine the gas content in the air, i.e., the total volume of bubbles formed when the pressure is reduced from elevated to atmospheric conditions. The excess pressure is generated by a pump supplying recirculation water to the saturator, and bubbles are formed in the flotation unit when the air-saturated recirculation water mixes with the wastewater.

The gas content in a mixture of wastewater and recirculated water can be calculated via one of the formulas proposed by the authors, Eq. (5) or Eq. (6).

5 $$\:{G}_{\left(i\right)}=\frac{273{k}_{s}{k}_{d}{W}_{\left(i\right)}\left({P}_{p}-{P}_{c}-{P}_{h}\right){q}_{rw}}{\mathrm{0,101335}(273+{t}_{air})({q}_{ww}+{q}_{rw})},\mathrm{c}\mathrm{m}^3/\mathrm{d}\mathrm{m}^3\:(\mathrm{d}\mathrm{m}^3/\mathrm{m}^3)$$

6 $$\:{G}_{\left(i\right)}=\frac{273{k}_{s}{k}_{d}{W}_{\left(i\right)}\left({P}_{s}-{P}_{h}\right){R}_{rw}}{\mathrm{0,101335}(273+{t}_{air})(1+{R}_{rw})},\mathrm{c}\mathrm{m}^3/\mathrm{d}\mathrm{m}^3\:(\mathrm{d}\mathrm{m}^3/\mathrm{m}^3)$$

where $\:{G}_{\left(i\right)}$ – is the gas content of the mixture of wastewater and recirculated water in air at the *i*-th water temperature, cm³/dm³ (dm³/m³); $\:{k}_{s}$ – is a coefficient depending on the type of saturator (based on the authors’ research, the following values can be assumed for a bubbling type saturator: - $\:{k}_{s}$ = 0.16…0.35, for a jet aeration saturator; – $\:{k}_{s}$ = 0.7…0.9, for a two-stage saturator; – $\:{k}_{s}$ = 0.8…0.95, for a saturator with a mass transfer nozzle; - $\:{k}_{s}$ = 0.95…1.0); $\:{k}_{d}$ – coefficient depending on the duration (*T*) of recirculating water stay in the saturator (can be taken for *T* = 2 min.); $\:{k}_{d}$ = 0.7…0.8, for T = 5 min.; $\:{k}_{d}$ = 0.8…0.9, for T > 5 min.; $\:{k}_{d}$ = 0.9…0.95; $\:{W}_{\left(i\right)}$ – maximum volume concentration of air in recirculation water at the *i*-th water temperature, taken from Table 2, cm³/dm³ (dm³/m³)); $\:{P}_{p}$ – pump pressure when supplying recirculation water to the saturator, MPa; $\:{P}_{c}$ – pressure losses in the communications from the pump to the saturator, MPa; $\:{P}_{h}$ – hydrostatic pressure at the level of mixing wastewater with recirculation water, MPa; $\:{P}_{s}={P}_{p}-{P}_{c}\:$ – pressure in the saturator, MPa; $\:{q}_{rw}\:$– hourly flow rate of recirculating water, m³/h; $\:{q}_{ww}$ – hourly flow rate of wastewater, m³/h; $\:{t}_{air}$ – air temperature, °C; $\:{R}_{rw}$ – recirculation ratio – the ratio of the hourly flow rate of recirculating water to the hourly flow rate of wastewater.

Equation (5) accounts for the pump pressure during the supply of recirculating water to the saturator, as well as the flow rates of wastewater and recirculated water, and is used to calculate the gas content on the basis of the operating characteristics of the pump. Equation (6), derived from Eq. (5), considers the pressure in the saturator and the recirculation ratio between the recirculation water flow rate and the wastewater flow rate. The key difference from Eq. (5) is that, instead of the individual flow rates, a dimensionless parameter–the recirculation ratio–is used. This allows Eq. (6) to be applied for calculating the gas content even when the wastewater flow rate is unknown.

**Fig. 1:**
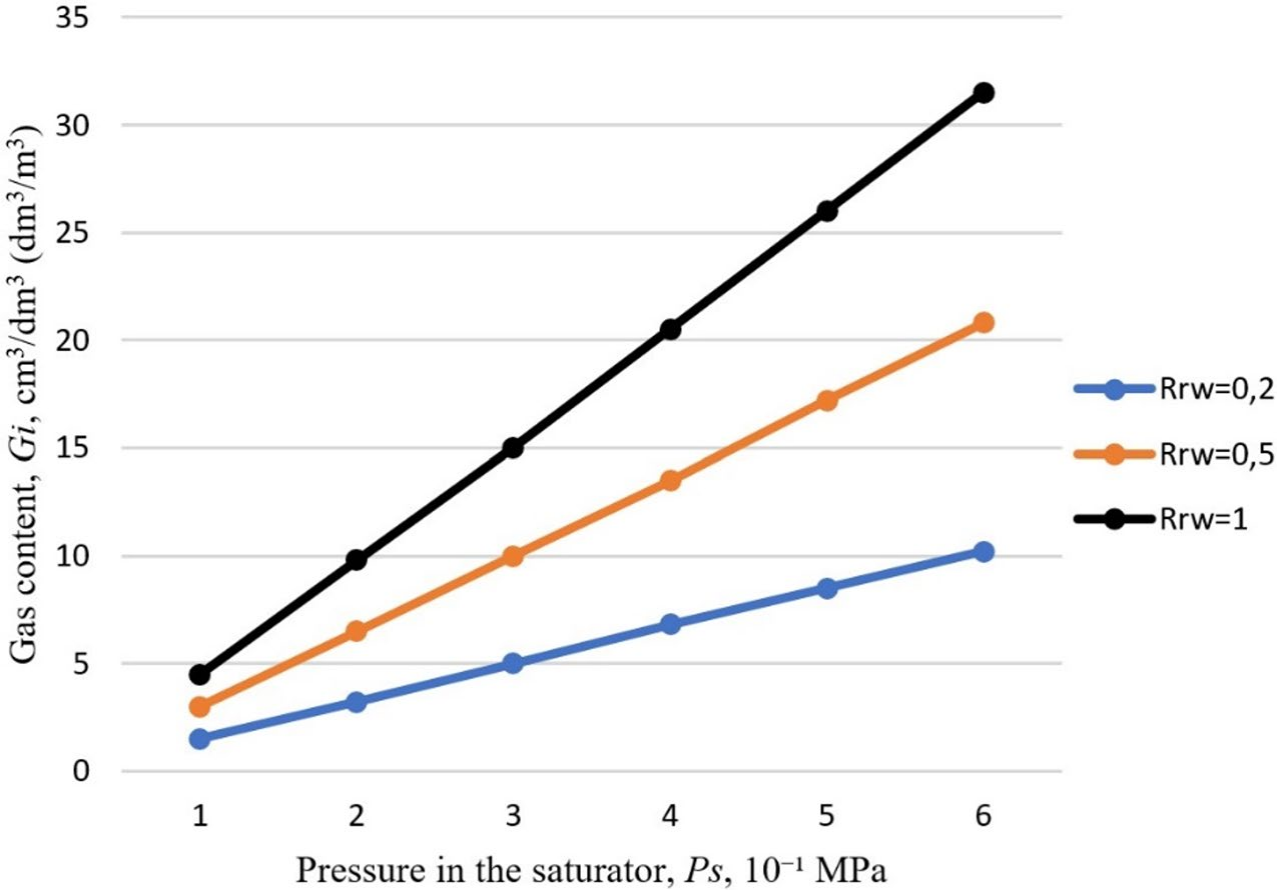
The gas content depends on the pressure in the saturator $\:{P}_{s}$ and the recirculation ratio $\:{R}_{rw}$ at a water temperature of 20 °C.

Figure 1 shows a graph calculated via formula (6) of the dependence of the gas content $\:{G}_{\left(i\right)}$ on the pressure in the saturator $\:{P}_{s}$ and recirculation ratio $\:{R}_{rw}$ at a water temperature of 20 °C, $\:{k}_{s}$=0.8, $\:{k}_{d}$ = 0.8, $\:{W}_{\left(i\right)}$ = 18.3 cm³/dm³ (dm³/m³) (Table 2), $\:{P}_{h}$ = 0.02 MPa, and $\:{t}_{air}$ = 200 °C.

As shown in the graphs (Fig. 1) and from calculations via Eq. (6), the change in gas content is not proportional to the saturator pressure $\:{P}_{s}$ alone but rather to the pressure difference $\:{P}_{s}-{P}_{h}$. For example, at a saturator pressure of 0.2 MPa and a recirculation ratio of $\:{R}_{rw}$= 0.5, the gas content is 6.46 cm³/dm³ (dm³/m³). When the pressure is increased to 0.4 MPa, the gas content increases 2.1–13.64 cm³/dm³ (dm³/m³), which, at $\:{P}_{h}$= 0.02 MPa, corresponds to the ratio ((0.4–0.02)/(0.2–0.02) = 2.1. Therefore, calculations must account for the effect of hydrostatic pressure $\:{P}_{h}$ on the gas content when mixing wastewater with recirculated water, particularly in high-altitude flotation plants.

The recirculation ratio $\:{R}_{rw}$ has a considerably smaller effect on the gas content than the saturator pressure. For example, doubling $\:{R}_{rw}$ from 0.5 to 1 at a saturator pressure of 0.4 MPa results in only a 1.5-fold increase in gas content, from 13.64 cm³/dm³ (dm³/m³) to 20.47 cm³/dm³ (dm³/m³). A higher gas content of 20.83 cm³/dm³ (dm³/m³) at $\:{R}_{rw}$ = 0.5 can be achieved by increasing the saturator pressure to 0.6 MPa, i.e., 1.5 times. From an energy perspective, increasing the saturator pressure $\:{P}_{s}$ is thus more economical than increasing the recirculation ratio $\:{R}_{rw}$.

Moreover, the total flow of the mixture of wastewater and recirculated water, as well as the degree of wastewater dilution, depends on $\:{R}_{rw}$. Significant dilution is required for flotation treatment of concentrated wastewater containing more than 1.0 g/dm³ of metals, in which case $\:{R}_{rw}$ ≥ 1 is typically assumed.

Since the resulting air-in-water emulsion is polydisperse, it is necessary to know the bubble size distribution. Taking this into account, the concentration of bubbles $\:{N}_{b\left(j\right)}$ with diameter $\:{D}_{b\left(j\right)}$ can be expressed as follows:

7 $$\:{N}_{b\left(j\right)}=\frac{{p}_{b\left(j\right)}{G}_{\left(i\right)}}{{w}_{b\left(j\right)}}=\frac{6{p}_{b\left(j\right)}{G}_{\left(i\right)}}{{10}^{-12}\pi\:{D}_{b\left(j\right)}^{3}},1/\mathrm{d}\mathrm{m}^3$$

where $\:{p}_{b\left(j\right)}$ is the proportion of bubbles with diameter $\:{D}_{b\left(j\right)}$; $\:{G}_{\left(i\right)}$ is the gas content of the mixture of wastewater and recirculated water by air at the *i*-th water temperature, cm³/dm³; $\:{w}_{b\left(j\right)}$ is the volume of a bubble with a diameter of $\:{D}_{b\left(j\right)}$, cm³; and $\:{D}_{b\left(j\right)}$ is the diameter of the bubbles for which their count concentration is determined, µm.

If needed, Eq. (7) can also be used to determine the number of nitrogen and oxygen bubbles separately after first calculating the corresponding gas content values via Eq. (5) or Eq. (6).

On the basis of the total bubble number concentration, it is possible to determine the volume of water associated with a single bubble, assuming that the bubbles are uniformly distributed in the mixture of wastewater and recirculated water:

8 $$\:{w}_{w}=1000/{\sum\:N}_{b\left(j\right)},\mathrm{c}\mathrm{m}^3$$

where $\:{w}_{w}$ is the volume of water containing one bubble, cm³, and where $\:{\sum\:N}_{b\left(j\right)}$ is the total countable concentration of bubbles in the mixture of wastewater and recirculating water, 1/dm³.

Under the same conditions, the distance between the centers of the bubbles can then be calculated as:

9 $$\:{l}_{b}=\sqrt[3]{{w}_{w}},\mathrm{c}\mathrm{m}$$

In practice, the distance between bubbles may be greater or smaller than the value calculated via Eq. (9) because of the random distribution of bubbles at the time of their formation and growth. Nevertheless, determining the distance between bubble centers provides essential preliminary information for analyzing the characteristics of wastewater treatment by pressure flotation.

Table 3 presents the values of $\:{N}_{b}$, $\:{w}_{w}$ and $\:{l}_{b}$ calculated via Eqs. (7)–(9) as a function of bubble diameter, assuming a nominal gas content of 1 cm³/dm³ of water. The calculated bubble concentration $\:{N}_{b}$ is used to analyze the behavior of the flotation process, whereas the distance between bubble centers $\:{l}_{b}\:$ is used to examine the patterns of bubble coalescence.

**Table 3:** Calculated values of $\:{N}_{b}$, $\:{w}_{w}$ and $\:{l}_{b}$ at $\:G$ = 1 cm³/dm³.

Calculated values	Bubble diameter, µm				
	20	40	60	80	100
$\:{N}_{b}$, 1/dm³	239 million	30 million	8,8 million	3,7 million	1,9 million
$\:{w}_{w}$, cm^3^	4,2$\:\cdot\:$10^−6^	3,3$\:\cdot\:$10^−5^	1,1$\:\cdot\:$10^−4^	2,7$\:\cdot\:10$^−4^	5,3$\:\cdot\:$10^−4^
$\:{l}_{b}$, cm (μm)	0,016 (160)	0,032 (320)	0,047 (470)	0,063 (630)	0,081 (810)

For gas content values other than $\:G$ = 1 cm³/dm³, the bubble number concentration $\:{N}_{b}$ changes proportionally relative to the values presented in Table 3. The maximum gas content in water can be achieved when the distance between the centers of the bubbles equals their diameter,$\:\:{(l}_{b}$ = $\:{D}_{b\left(j\right)})$.

This condition corresponds to all the bubbles being in contact with each other. Considering this, the minimum volume of water ($\:{w}_{w\left(min\right)})$, containing a single bubble in a cubic packing arrangement, can be calculated as:

10 $$\:{w}_{w\left(min\right)}={10}^{-12}{D}_{b\left(j\right)}^{3},\:\mathrm{c}\mathrm{m}^3$$

The total maximum bubble number concentration ($\:{\sum\:N}_{b\left(max\right)}$) and the maximum gas content ($\:{G}_{max}$) can be expressed as:

11 $$\:{\sum\:N}_{b\left(max\right)}=1000/{w}_{w\left(min\right)}={10}^{15}/{D}_{b\left(j\right)}^{3},\:1/\mathrm{d}\mathrm{m}^3$$

12 $$\:{G}_{max}={\sum\:N}_{b\left(max\right)}{w}_{b\left(j\right)}=\frac{{10}^{15}\pi\:{D}_{b\left(j\right)}^{3}}{6{\cdot10}^{12}{D}_{b\left(j\right)}^{3}}=1000\pi\:/6=523\:\mathrm{c}\mathrm{m}^3/\mathrm{d}\mathrm{m}^3$$

Thus, the maximum possible gas content is independent of bubble size and, assuming cubic packing of the bubbles, equals 523 cm³/dm³, or 52.3%.

During bubble rise, coalescence occurs, leading to an increase in bubble volume and diameter and a corresponding decrease in bubble number concentration. The bubble volume, $\:{w}_{b\left(c\right)}$, and bubble diameter, $\:{D}_{b\left(c\right)}$, after coalescence can then be calculated via the following formulas:

13 $$\:{w}_{b\left(c\right)}=\sum\:\left({n}_{\left(j\right)}{w}_{b\left(j\right)}\right),\:\mathrm{c}\mathrm{m}^3$$

14 $$\:{D}_{b\left(c\right)}=\sqrt[3]{{\sum\:({n}_{\left(j\right)}D}_{b\left(j\right)}^{3})\:\:,\:{\upmu\:}\mathrm{m}}$$

where $\:{n}_{\left(j\right)}$ is the number of coalescing bubbles with an initial volume $\:{w}_{b\left(j\right)}$ and diameter $\:{D}_{b\left(j\right)}$, pcs. where $\:{w}_{b\left(j\right)}$ is the initial volume of the *j*-th bubble, cm³, and $\:{D}_{b\left(j\right)}$ is the initial diameter of the *j*-th bubble, µm.

The efficiency of the flotation process depends on the ratio of the bubble number concentration to the particle number concentration, which can be expressed by Eq. (15):

15 $$\:{\sum\:N}_{p\left(i\right)}=\sum\:\left({C}_{p\left(i\right)}/{m}_{p\left(i\right)}\right)={\sum\:(C}_{p\left(i\right)}/{1000w}_{p\left(i\right)}{\rho\:}_{p\left(i\right)}),1/\mathrm{d}\mathrm{m}^3$$

where $\:{\sum\:N}_{p\left(i\right)}$ is the total count concentration of particles in the mixture of wastewater and recirculated water, 1/dm^3^; $\:{C}_{p\left(i\right)}$ is the concentration of particles with volume $\:{w}_{p\left(i\right)}$ and density $\:{\rho\:}_{p\left(i\right)}$, mg/dm^3^; $\:{m}_{\mu\left(i\right)}$ is the mass of the i-th particle, mg; $\:{w}_{p\left(i\right)}\:$is the volume of the *i*-th particle, cm^3^; and $\:{\rho\:}_{p\left(i\right)}$ is the density of the *i*-th particle, g/cm^3^.

When floating macrodisperse particles, such as metal hydroxides, the number of bubbles must exceed the number concentration of the particles:

16 $$\:\sum\:{N}_{b\left(j\right)}>{\sum\:N}_{p\left(i\right)},\:1/\mathrm{d}\mathrm{m}^3$$

From inequality (16), it follows that $\:\sum\:{N}_{b\left(j\right)}/{\sum\:N}_{p\left(i\right)}>1$; that is, the gas‒solid particle ratio based on the number concentrations of bubbles and particles must be greater than 1. Moreover, the G/S ratio, which is based on the mass concentrations of air and particles obtained from experimental data, is less than 1 (G/S < 1)^11,12^. For practical calculations, the experimentally determined G/S ratio is used, whereas the ratio $\:\sum\:{N}_{b\left(j\right)}/{\sum\:N}_{p\left(i\right)}$ is employed for analyzing the theoretical patterns of the pressure flotation process.

For bubbles with a weighted average volume $\:{w}_{b\left(av\right)}$ and particles with a weighted average volume $\:{w}_{p\left(av\right)\:}$ and density $\:{\rho\:}_{p\left(av\right)}$, the following equations can be derived:

17 $$\:\frac{{G}_{\left(i\right)}}{{w}_{b\left(av\right)}}>\frac{{C}_{p\left(mix\right)}}{{1000w}_{p\left(av\right)}{\rho\:}_{p\left(av\right)}},\:1/\mathrm{d}\mathrm{m}^3$$

18 $$\:{C}_{p\left(mix\right)}<\frac{1000{{G}_{\left(i\right)}{w}_{p\left(av\right)}\rho\:}_{p\left(av\right)}}{{w}_{b\left(av\right)}},\:\mathrm{m}\mathrm{g}/\mathrm{d}\mathrm{m}^3$$

where $\:{G}_{\left(i\right)}$ is the gas content determined by Eq. (5) or (6), or experimentally, cm³/dm³; $\:{C}_{p\left(mix\right)}$ is the concentration of particles in the mixture of wastewater and recirculated water, mg/dm³;

19 $$\:{C}_{p\left(mix\right)}=\frac{{C}_{p\left(ww\right)}{q}_{ww}+{C}_{p\left(rw\right)}{q}_{rw}}{{q}_{ww}+{q}_{rw}}=\:\frac{{C}_{p\left(ww\right)}+{R}_{rw}{C}_{p\left(rw\right)}}{(1+{R}_{rw})},\:\mathrm{m}\mathrm{g}/\mathrm{d}\mathrm{m}^3\:(\mathrm{g}/\mathrm{m}^3)$$

where $\:{C}_{p\left(ww\right)}\:$ is the concentration of particles in wastewater, mg/dm^3^, and where $\:{C}_{p\left(rw\right)}\:$ is the concentration of particles in recirculated water, mg/dm^3^.

From inequality (18), it follows that, at a constant gas content, the permissible particle concentration – at which the number of bubbles exceeds the number of particles – depends primarily on the ratio of the volumes of particles and bubbles. For example, at a gas content of 50 cm³/dm³, a weighted average bubble diameter of 50 μm, a weighted average particle density of 1.5 g/cm³, and a weighted average particle diameter of 1 μm, the particle concentration must be less than 0.6 mg/dm³, whereas for a particle diameter of 10 μm, it must be less than 600 mg/dm³.

An analysis of inequality (18) indicates that since the volume of each flake-like metal hydroxide particle exceeds the volume of each bubble formed during pressure flotation, the number of bubbles will be much greater than the number of particles at any realistic total metal concentration in wastewater. Therefore, the most likely scenario is the formation of flotation complexes consisting of one particle and one or more air bubbles. In this case, the number of bubbles per particle, ($\:{n}_{b}$), can be expressed as:

20 $$\:{n}_{b}=\frac{\sum\:{N}_{b\left(j\right)}}{{\sum\:N}_{p\left(i\right)}}=\frac{1000{{G}_{\left(i\right)}\rho\:}_{p\left(av\right)}}{{C}_{p\left(mix\right)}}\cdot\frac{{w}_{p\left(av\right)}}{{w}_{b\left(av\right)}}={K}_{b\left(gen\right)}\cdot{K}_{p\left(av\right)}$$

where $\:{K}_{b\left(gen\right)}$ is a dimensionless total volume criterion—the ratio of the total volume of bubbles to the total volume of particles—and where $\:{K}_{p\left(av\right)}$ is a dimensionless weighted average volume criterion—the ratio of the weighted average volume of one particle to the weighted average volume of one bubble.

The number of bubbles per particle, ($\:{n}_{b})$, must exceed the number of bubbles required for flotation complexes to rise, $\:({n}_{b\left(fl\right)}$):

21 $$\:{n}_{b}>{n}_{b\left(fl\right)}$$

A flotation complex floats if its density ($\:{\rho\:}_{fl})$ is less than the density of water ($\:{\rho\:}_{ww})$:

22 $$\:{\rho\:}_{fl}<{\rho\:}_{ww}$$

The density of a flotation complex, considering the attachment of multiple bubbles of average volume to a single particle of average volume, can be calculated as follows:

23 $$\:{\rho\:}_{fl}=\frac{{m}_{p\left(av\right)}+{{n}_{b\left(fl\right)}m}_{b\left(av\right)}}{{w}_{p\left(av\right)}+{{n}_{b\left(fl\right)}w}_{b\left(av\right)}}=\frac{{w}_{p\left(av\right)}{\rho\:}_{p\left(av\right)}}{{w}_{p\left(av\right)}+{{n}_{b\left(fl\right)}w}_{b\left(av\right)}}=\frac{{\rho\:}_{p\left(av\right)}}{1+{n}_{b\left(fl\right)}\frac{{w}_{b\left(av\right)}}{{w}_{p\left(av\right)}}},\:\mathrm{g}/\mathrm{c}\mathrm{m}^3$$

where $\:{m}_{p\left(av\right)}$ is the mass of a particle of average volume, g; $\:{m}_{b\left(av\right)}$ is the mass of a bubble of average volume, g; and $\:{\rho\:}_{p\left(av\right)}$ is the density of a particle, g/cm^3^.

The mass of the bubble is negligible and is therefore not considered when calculating the density of the flotation complex. Inequality (22), taking into account expression (23), can be written as follows:

24 $$\:\frac{{\rho\:}_{p\left(av\right)}}{1+{n}_{b\left(fl\right)}\frac{{w}_{b\left(av\right)}}{{w}_{p\left(av\right)}}}<{\rho\:}_{ww}$$

We obtain the number of bubbles needed for the flotation complexes to float ($\:{n}_{b\left(fl\right)}$):

25 $$\:{n}_{b\left(fl\right)}>(\frac{{\rho\:}_{p\left(av\right)}}{{\rho\:}_{ww}}-1)\:\frac{{w}_{p\left(av\right)}}{{w}_{b\left(av\right)}}>(\frac{{\rho\:}_{p\left(av\right)}}{{\rho\:}_{ww}}-1)\:{K}_{p\left(av\right)}$$

From Eq. (25), if the volume of a particle significantly exceeds the volume of a bubble, then one or more bubbles must be attached to it to achieve flotation of a high-density particle. In the case where flotation occurs with at least one bubble attached to the particle ($\:{n}_{b\left(fl\right)}$=1), the particle density, ($\:{\rho\:}_{p\left(1\right)}$), must be less than the following value:

26 $$\:{\rho\:}_{p\left(1\right)}<{\rho\:}_{ww}(1+\frac{{w}_{b\left(av\right)}}{{w}_{p\left(av\right)}})<{\rho\:}_{ww}(1+\frac{1}{{K}_{p\left(av\right)}}),\:\mathrm{g}/\mathrm{c}\mathrm{m}^3$$

Table 4 shows the calculated values of particle density ($\:{\rho\:}_{p\left(1\right)}$) for which flotation can be achieved with a single bubble, depending on the weighted average volume criterion $\:{K}_{p\left(av\right)}$ at a wastewater density of $\:{\rho\:}_{ww}=$0.998 g/cm^3^ (temperature 20 °C).

**Table 4:** Calculated permissible particle density ($\:{\rho\:}_{p\left(1\right)}$) for single-bubble flotation depending on the weighted average volume criterion $\:{K}_{p\left(av\right)}$ at a wastewater temperature of 20 °C.

$\:{\boldsymbol{K}}_{\boldsymbol{p}\left(\boldsymbol{a}\boldsymbol{v}\right)}$	2	5	10	20	50
$\:{\rho\:}_{p\left(1\right)}$, g/сm^3^	less than 1,497	less than 1,198	less than 1,098	less than 1,048	less than 1,018

As is well known, particles of most metal hydroxides have a loose, porous structure, with cavities filled with water. Consequently, the density of hydroxide flakes is significantly lower than that of fully crystalline particles of the same hydroxides.

The density of metal hydroxide flakes ($\:{\rho\:}_{fs}$), taking into account the proportion of solid matter within their structure, can be calculated via the following equation^20^:

27 $$\:{\rho\:}_{fs}={\rho\:}_{ww}+\gamma\:({\rho\:}_{s}-{\rho\:}_{ww}),\:\mathrm{g}/\mathrm{c}\mathrm{m}^3$$

where $\:{\rho\:}_{s}$ is the density of the solid phase in the flake structure, g/cm^3^, and *γ* is the volume of solid matter per unit volume of flakes, cm^3^/cm^3^.

Table 5 shows the density of the solid phase of some metal hydroxides at a temperature of 20 °C^21^.

**Table 5:** Density of the solid phase of metal hydroxides^21^.

Metal hydroxide	Al(OH)_3_	Fe(OH)_2_	Fe(OH)_3_	Zn(OH)_2_	Cu(OH)_2_	Cr(OH)_3_	Ni(OH)_2_
Density, g/cm^3^	2,42	3,40	3,0–3,12	3,03	3,358	2,90	3,65

The solid-phase content of hydroxides, with the densities given in Table 5, in the flaky structure of floatable particles determines the density of these particles. For example, in loose aluminum hydroxide particles, the volume of crystalline substance is relatively small, measured in tens of percent. Consequently, the density of aluminum hydroxide flakes is 1.001–1.003 g/cm³^20^, which is close to the density of water. When suspended solids are present in wastewater, the density of aluminum hydroxide flakes increases to 1.01–1.03 g/cm³^20^.

A comparison of these density values with the particle densities in Table 4 reveals that aluminum hydroxide flakes can be floated by a single bubble even at $\:{K}_{p\left(av\right)}$= 50 (the particle volume is 50 times greater than the bubble volume). Given the density of aluminum hydroxide flakes ($\:{\rho\:}_{fs}$ = 1.001–1.003 g/cm³), the volume of solid matter per unit volume of these flakes (the specific volume of solid matter) can be determined by rearranging Eq. (27):

28 $$\:\gamma\:=({\rho\:}_{fs}-{\rho\:}_{ww})/({\rho\:}_{s}-{\rho\:}_{ww}),\:\mathrm{c}\mathrm{m}^3/\mathrm{c}\mathrm{m}^3$$

The calculations via Eq. (28) reveal that at a wastewater temperature of 20 °C ($\:{\rho\:}_{ww}\:$= 0.998 g/cm³) and a solid-phase density of Al(OH)₃ ($\:{{\uprho\:}}_{\mathrm{s}}$ = 2.42 g/cm³), the specific volume of solid matter within aluminum hydroxide flakes is 0.002–0.004 cm³/cm³, or 0.2–0.4%.

For the same specific volume of solid matter, nickel hydroxide flakes have a density of 1.003–1.009 g/cm³. Considering that the solid-phase densities of hydroxides of other metals are lower than those of nickel hydroxide ($\:{{\uprho\:}}_{\mathrm{s}}$ = 3.45 g/cm³, Table 5), the densities of the corresponding flake-like particles of these metals are lower than those of nickel hydroxide flakes with the same solid-phase fraction.

As a result, there is a theoretical possibility of floating individual metal hydroxide flakes via a single bubble. Therefore, the formation of flotation complexes is highly probable at the gas contents typically used in flotation plants and, correspondingly, at practical gas-to-solid particle ratios (*G/S*). The *G/S* ratio for metal-containing wastewater is determined experimentally since calculating it from the theoretical dependencies requires knowledge of the number concentration, size, and density of flake-shaped metal hydroxide particles.

On the basis of the experimental *G/S* value, the recirculation water flow rate can be calculated for different metal concentrations in wastewater. To do this, the required gas content ($\:{G}_{i}$) in the flotation chamber must first be determined:

29 $$\:{G}_{i}=\frac{G}{S}\cdot\:\frac{{C}_{m}}{{\rho\:}_{air}},\:\mathrm{c}\mathrm{m}^3/\mathrm{d}\mathrm{m}^3\:(\mathrm{d}\mathrm{m}^3/\mathrm{m}^3)$$

where G/S is the experimentally determined gas‒solid ratio; $\:{C}_{m}$ is the concentration of metals in wastewater, mg/dm^3^ (g/m^3^); and $\:{\rho\:}_{air}$ is the density of air, which depends on temperature, pressure and humidity, taken from reference data, mg/cm^3^ (g/dm^3^).

According to Eq. (29), the gas content ($\:{G}_{i}$) is directly proportional to the total metal concentration in the wastewater, assuming a constant experimentally determined *G/S* ratio. After determining ($\:{G}_{i}$) for the maximum possible metal concentration in the wastewater of a given production facility, the recirculation water flow rate, $\:{q}_{rw}$, can be calculated by rearranging Eq. (5), or the recirculation ratio can be calculated by rearranging Eq. (6):

30 $$\:{q}_{rw}=\frac{\mathrm{0,101335}(273+{t}_{air}){{G}_{\left(i\right)}q}_{ww}}{273{k}_{s}{k}_{d}{W}_{\left(i\right)}\left({P}_{p}-{P}_{c}-{P}_{h}\right)-\mathrm{0,101335}(273+{t}_{air}){G}_{\left(i\right)}},\:\mathrm{m}^3/\mathrm{h}$$

31 $$\:{R}_{rw}=\frac{\mathrm{0,101335}(273+{t}_{air}){G}_{\left(i\right)}}{273{k}_{s}{k}_{d}{W}_{\left(i\right)}\left({P}_{s}-{P}_{h}\right)-\mathrm{0,101335}(273+{t}_{air}){G}_{\left(i\right)}}$$

When calculating $\:{q}_{rw}$ or $\:{R}_{rw}$, the pressure of the pump for supplying recirculating water to the saturator is taken to be within the range of 0.3–0.6 MPa.

After determining $\:{q}_{rw}$ or $\:{R}_{rw}$, the volume of the saturator is calculated on the basis of the residence time of the recirculating water and the dimensions of the flotation unit. The methodology for experimentally determining the parameters of the flotation process for metal extraction, including the *G/*S ratio, is presented in the authors’ work^12^.

Thus, research has demonstrated that when pressure flotation is used to treat metal-containing water, the efficiency of the process strongly depends on the ratio of the concentration of insoluble metal compounds to the number of air bubbles. The calculations indicate that since the volume of each flake-like particle formed during alkalization exceeds the volume of each bubble released from the air-saturated recirculating water, several bubbles are present per particle under real conditions. Moreover, the formation of flotation complexes consisting of just one particle and one bubble is sufficient for flotation. This is due to the loose structure of the particles of poorly soluble metal compounds, whose cavities contain bound water, resulting in flakes with a density close to that of water.

The efficiency of treating metal-containing wastewater by pressure flotation is strongly influenced by the conditions under which a flaky structure forms after metals are converted into poorly soluble compounds. Bench-scale studies have shown that flotation kinetics and surface interactions significantly influence microplastic removal efficiency, with parameters such as particle density, size, and organic substances in solution altering flotation performance^22^. Similarly, in the present study, the more developed the flaky structure is, the lower the optimal *G/S* ratio and, consequently, the lower the energy consumption of the process.

Therefore, to form a loose flake structure, the coagulation of poorly soluble metal compounds should be carried out in the presence of bubbles generated in the flotation unit. In this case, the bubbles become incorporated into the metal hydroxide flakes, reducing their density below that of water and enabling effective flotation. This approach not only lowers the energy consumption of the flotation process but also allows for fractional dosing of alkali to selectively extract metals from wastewater for subsequent disposal.

Similar approaches involving surface hydrophobic modification and flotation-based particle separation have been successfully applied in other contexts, such as high-sulfur coal desulfurization and gasification fine slag treatment^23,24^. These studies demonstrate that controlling bubble‒particle interactions and optimizing flotation conditions can significantly increase the removal of target compounds. The findings reported by Cheng et al.^23,24^support the broader applicability of controlled flotation processes, highlighting the relevance of such methods beyond wastewater treatment and metal extraction and providing a comparative basis for the methodology applied in the present study.

### Two-stage flotation unit and experimental results

For wastewater containing a mixture of metals with different pH values for hydroxide formation, the authors developed a two-stage flotation unit for selective metal extraction, which is currently used in several enterprises across European countries (Fig. 3).

A characteristic feature of this flotation unit is that all structural components necessary for effective selective treatment of metal-containing water are integrated into a single module. The flotation unit is equipped with a common air-saturation system for water recirculation, comprising a flow-through saturator, a high-pressure pump, and an ejector for saturating the recirculated water with dissolved air. In addition, each flotation stage is equipped with a mixer, a flocculator, individual recirculated-water dosing systems, a flow controller, automatic pH control systems based on proportional–integral–derivative (PID) controllers with parametric optimization, and a device for automatic sludge removal.

**Fig. 2:**
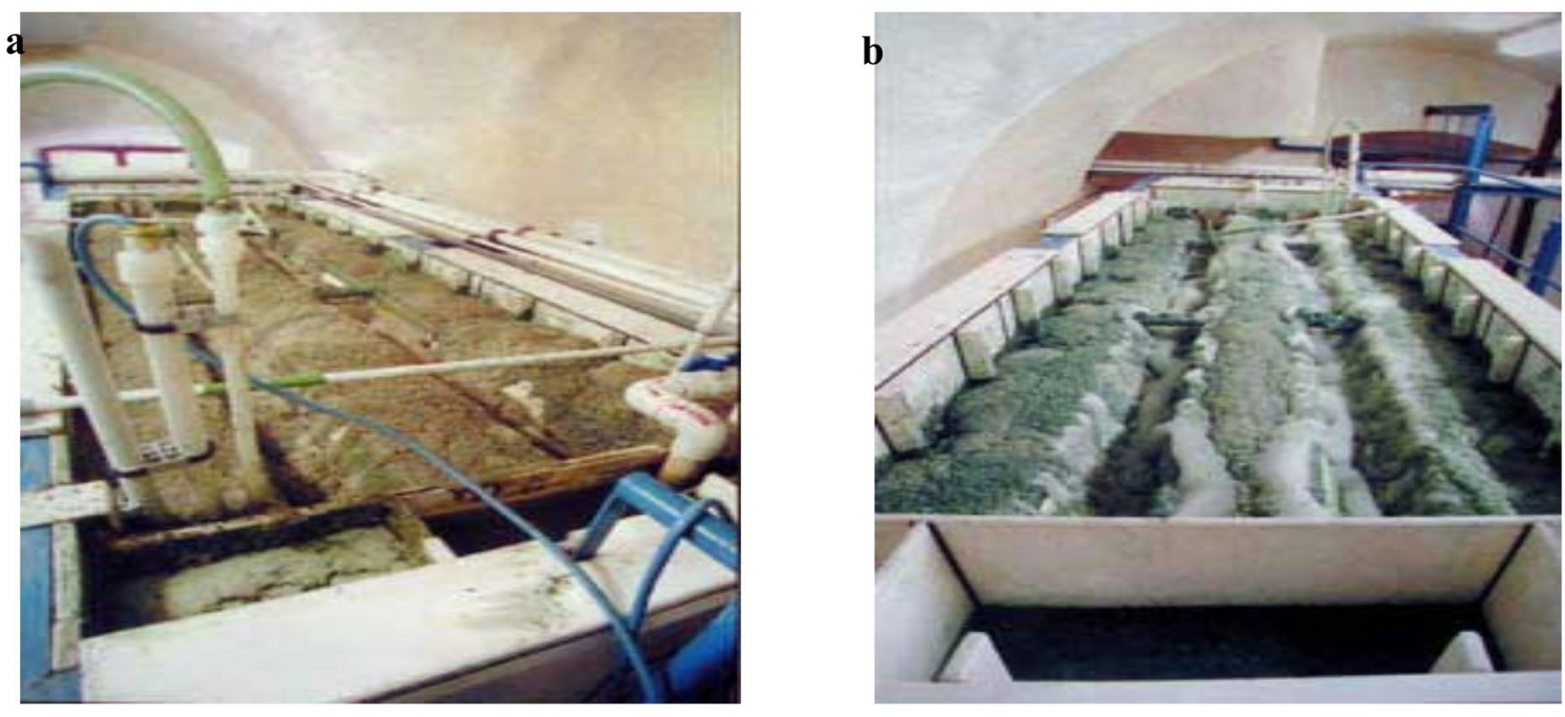
Two-stage flotation unit with a capacity of 15 m^3^/h for the selective extraction of metals with different pH hydroxide formations from electroplating wastewater (developed by the authors): a – first stage of the flotation unit; b – second stage of the flotation unit.

This made it possible to float metal hydroxides with varying hydrate formation pH values. The first stage enables the extraction of Fe(III), Sn, and Cr(III) ions, as well as amphoteric metals such as Al and Zn, which have hydration pH values in the range of 4.1–8.0. The second stage allows for the extraction of Fe(II), Ni, and Cd ions, with a hydration pH of 9.2–9.7. As shown in Fig. 2, the flotation sludge of the metal hydroxides formed at each stage, corresponding to different hydration pH values, exhibited distinct colors.

Table 6 presents the results of studies on the treatment of metal-containing water via pressure flotation in a two-stage flotation unit with a capacity of 15 m³/h. During the tests, the recirculated water flow rate (recirculation ratio) was varied while maintaining a constant pressure in the saturator of 0.4 MPa. The pH in the first flotation stage ranged from 7.7 to 8.2, whereas in the second stage, it ranged from 9.5 to 9.7. The temperature of the treated water varied between 19 and 21 °C, and the air temperature ranged from 20.2 to 20.4 °C. Downstream of the saturator, the recirculated water stream was supplied to the first and second flotation chambers of the unit in equal amounts (q_rw_/2).

**Table 6:** Results of studies on the treatment of metal-containing water by pressure flotation in a two-stage flotation unit with a capacity of 15 m³/h.

Metal	$\:{\boldsymbol{q}}_{\boldsymbol{r}\boldsymbol{w}}$ m^3^/h	$\:{\boldsymbol{R}}_{\boldsymbol{r}\boldsymbol{w}}$	$\:{\boldsymbol{G}}_{\boldsymbol{i}}$ dm^3^/m^3^	$\:{\boldsymbol{C}}_{0\left(\boldsymbol{m}\right)}$ mg/l	$\:{\boldsymbol{C}}_{0\left(\boldsymbol{h}\boldsymbol{m}\right)}$ mg/l	$\:{\boldsymbol{C}}_{1\left(\boldsymbol{m}\right)}$ mg/l	$\:{\boldsymbol{C}}_{2\left(\boldsymbol{m}\right)}$ mg/l
Iron (Fe^3+^)	3,0	0,2	6,8	112–146	214–279	33–42	27–34
	4,5	0,3	9,4			7,3–9,4	4,1–4,6
	7,5	0,5	13,6			6,9–9,1	3,9 − 4,2
Zinc (Zn^2+^)	3,0	0,2	6,8	83–97	126–148	13–19	9,5–11,3
	4,5	0,3	9,4			2,3 − 2,6	1,9 − 2,2
	7,5	0,5	13,6			2,1–2,4	1,8 − 2,1
Iron (Fe^2+^)	3,0	0,2	6,8	98–124	157–199	86–94	17–21
	4,5	0,3	9,4			78–85	2,4 − 2,9
	7,5	0,5	13,6			69–77	2,1–2,8
Nickel (Ni^2+^)	3,0	0,2	6,8	26–32	41–50	24–29	6,3–7,2
	4,5	0,3	9,4			17–24	0,8 − 1,3
	7,5	0,5	13,6			14–22	0,8 − 1,1

Legend: q_rw_ – total recirculated water flow rate; R_rw_ – recirculation ratio; G_i_ – total gas content; C_₀(m)_ – metal concentration in the wastewater; C_₀(hm)_ – concentration of suspended solids (metal hydroxide) in the wastewater; C_₁(m)_ – metal concentration in the water after the first flotation stage; C_₂(m)_ – metal concentration in the water after the second flotation stage.

According to the obtained results, the effective recirculation ratio for the tested metal-containing waters is 0.3, or the total gas content is 9.4 dm³/m³, since an increase in these parameters has virtually no effect on the metal concentration in the treated water.

At a total concentration of suspended solids (metal hydroxides) in the treated water of 538–676 mg/L, the average concentration is 607 mg/L (g/m³). Under these conditions, the calculated value of the gas-to-solids particle ratio (G/S) at an air temperature of 20 °C and an atmospheric pressure of 100 kPa (ρ_air_ = 1.188 g/dm³), according to Eq. (29), is as follows:

32 $$\:G/S={G}_{i}{\rho\:}_{air}/{C}_{0\left(hm\right)}=7,4\cdot1,188/607=0,014$$

The obtained value of the G/S ratio (G/S = 0.014) is therefore significantly lower than the values of this indicator (G/S = 0.02–0.06) reported for wastewater treated in a single-stage flotation unit^13^. Consequently, the energy consumption for treating metal-containing water via a two-chamber flotation unit is 1.4–4.3 times lower than that required for treating wastewater with suspended solids via a single-stage flotation unit while achieving a higher quality of metal removal.

A multistage pressure flotation system is particularly suitable when the total metal concentration in wastewater exceeds 500 mg/dm³. Under such conditions, selective metal recovery for recycling becomes economically viable. For example, with a metal concentration of 1.0 g/dm³ (1.0 kg/m³) and a flotation machine capacity of 15 m³/h, more than 60 tonnes of metals, including nonferrous metals, can be selectively extracted and recycled annually. The recycling of metals eliminates the need to dispose of dewatered flotation sludge in industrial waste landfills.

The experimental data presented in Table 6 and the calculated G/S ratio of 0.014 provide a new perspective on the operational efficiency of multi-chamber flotation units. Our research demonstrates that the high efficiency of pressure flotation in treating metal-containing water is primarily governed by the optimized interaction between air bubbles and the specific structure of the sediment particles.

As observed in our technical-scale tests, even a minimal number of bubbles per particle is sufficient to form stable flotation complexes. This phenomenon is attributed to the loose, flake-like structure of metal hydroxides (Fe, Zn, Ni) formed in our unit. These flocs contain bound water, which maintains their density close to that of the aqueous medium, facilitating rapid flotation even at low gas concentrations. While authors like Pooja et al. (2022)^25^ and Sztefek (2019)^26^ have emphasized the general importance of bubble size and pressure control, our findings provide specific quantitative evidence (Eq. 32) that a multi-chamber configuration allows for a significant reduction in gas requirements compared to traditional single-stage DAF systems.

A comparative analysis reveals a significant technological advantage of the proposed method. Pooja et al. (2021)^27^ achieved removal efficiencies exceeding 97% for ions such as Cr, Cd, and Ni, but their process required the addition of surface-active collectors (SDS) to ensure hydrophobicity. In contrast, our research proves that similar or superior removal efficiencies (reaching 98–99% for Nickel and Iron, as shown in Table 6) can be achieved without any surfactants. By leveraging the natural porous structure of the hydroxide flocs and precise pH control in the multi-chamber unit (7.7–9.7), we have eliminated the need for additional chemical reagents.

Furthermore, while literature^27^ confirms that flotation kinetics typically follow a first-order model dependent on bubble–particle collisions, our study demonstrates that the multi-chamber design actively enhances these kinetics. By separating the process into stages, we maximize the probability of effective collisions without increasing energy input. This is substantiated by our results showing a 1.4–4.3 times lower energy consumption than that required for single-stage units.

The proposed technological solutions, validated through technical-scale operation, enable not only high wastewater treatment efficiency but also the economically viable recovery of metals. With a capacity of 15 m^3^/h and concentrations exceeding 1.0 g/dm^3^, the selective extraction of over 60 tonnes of metals annually becomes possible. This approach directly aligns with the circular economy principles by transforming toxic wastewater treatment into a resource recovery process, effectively eliminating the environmental burden of sludge landfilling.

## Limitations and future perspectives

This research provides a coherent description of the mechanism of dissolved air flotation in the treatment of water containing metals and enables the identification of key parameters affecting separation efficiency. As in most engineering-type analyses, certain simplifying assumptions were adopted in this work, allowing for a clear representation of complex physicochemical phenomena without diminishing the cognitive or practical value of the obtained results. In this study, average values of particle and air bubble parameters, such as volume, diameter, and density, were used. Although real flotation systems are characterized by polydispersity, the adoption of mean values is a common and well-justified practice in flotation process modeling. It allows for the description of the dominant mechanisms of flotation complex formation and for the formulation of design relationships that can be directly applied in technological practice.

Another important aspect is the necessity of determining the *G/S* ratio experimentally for a specific type of wastewater. This results from the variability of industrial wastewater composition and the properties of the formed metal hydroxide precipitates. This approach was deliberately adopted in the present research, as it reflects real operational conditions of flotation installations and additionally enables flexible adjustment of process parameters to changing technological conditions. In this context, the presented analytical relationships serve as a supporting tool for the interpretation of results and the optimization of system performance.

Consequently, an important direction for further development is the continuation of research aimed at a more in-depth analysis of the mechanisms governing the formation of a loose, flocculent structure of metal hydroxides in the presence of air bubbles. Importantly, the influence of the process operating conditions, mixing intensity, and reagent dosing sequence on the structure and density of the resulting flocs must be determined. This is crucial, as it may enable a further reduction in the required G/S ratio and, consequently, a decrease in the energy consumption of the process. Another significant research area is the development and optimization of multistage flotation systems for the selective recovery of metals from multicomponent mixtures. Accordingly, future studies should include an evaluation of metal separation efficiency under varying concentrations and in the presence of cocontaminants.

Equally important are studies that focus on quality assessment and potential further utilization of the obtained flotation sludges. Another key direction for future research involves combining dissolved air flotation with other treatment processes for various contaminants, such as enhanced coagulation, membrane processes, or electrochemical methods, to expand the applicability of the developed technology and adapt it to increasingly stringent environmental requirements. Incorporating energy, environmental, and economic analyses at the industrial scale will allow for a more comprehensive assessment of the potential of dissolved air flotation as a technology compliant with the principles of sustainable development and the requirements of the best available techniques.

## Research methods

**Fig. 3:**
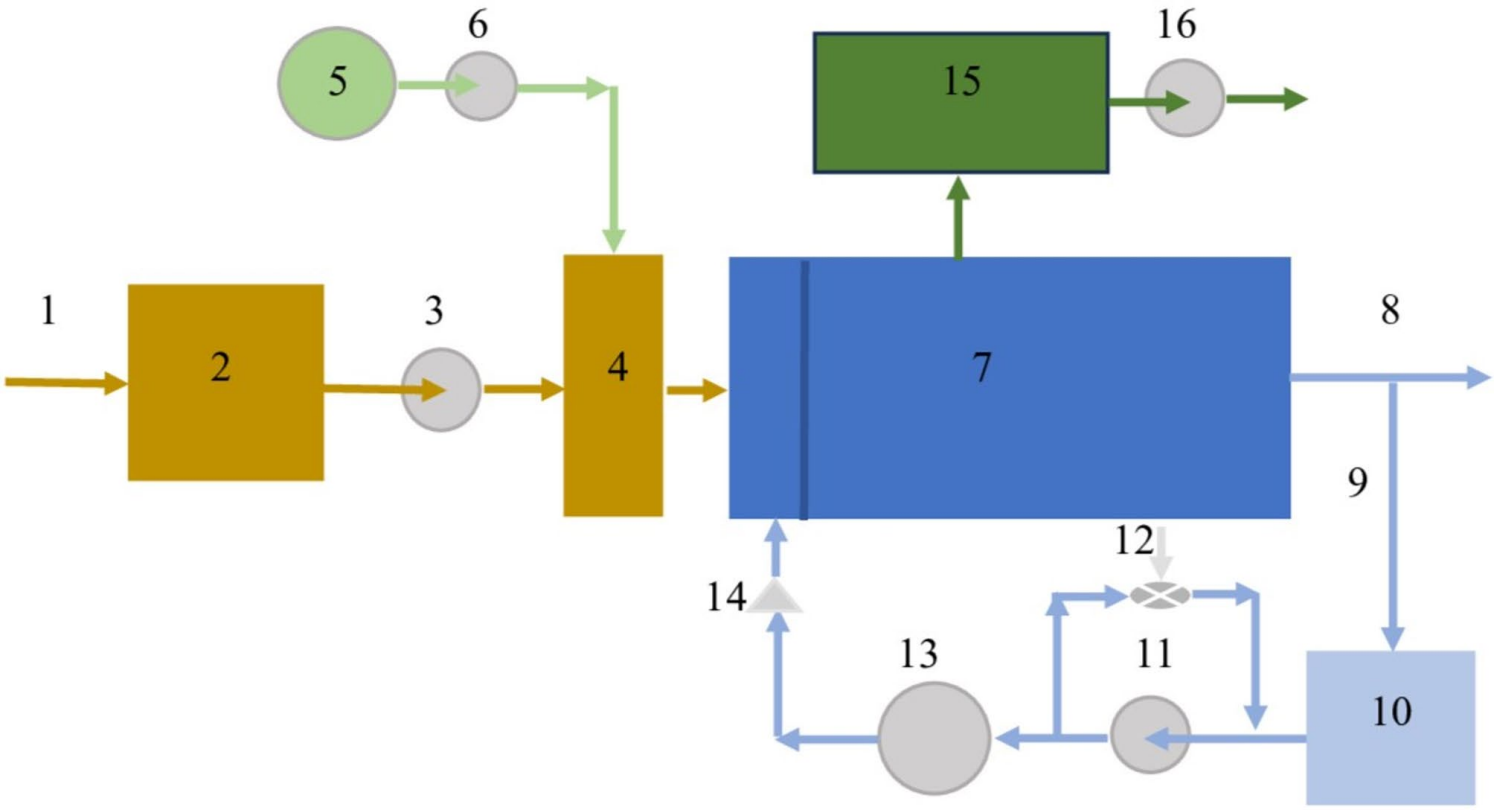
Process flow diagram of metal-containing wastewater treatment by pressure flotation: 1 – metal-containing wastewater; 2 – equalization tank (accumulator); 3 – pump for feeding wastewater to the treatment system; 4 – mixer; 5 – alkali solution storage tank; 6 – alkali solution dosing pump; 7 – flotation unit; 8 – treated water; 9 – recirculation water; 10 – recirculation water tank; 11 – pump supplying recirculation water to the saturator; 12 – ejector for air injection into the recirculation water; 13 – air saturator for dissolving air components (nitrogen and oxygen) under elevated pressure; 14 – throttling device for reducing pressure from elevated to atmospheric; 15 – flotation sludge storage tank; 16 – pump for conveying flotation sludge to dewatering.

To analyze the effects of gas content, concentration, particle size, and density on flotation performance, the most effective pressure flotation scheme developed by the authors, shown in Fig. 3, was employed.

The treatment process involves increasing the pH of the wastewater to a level at which poorly soluble metal compounds are formed. A portion of the treated (recirculated) wastewater is subsequently saturated with pressurized air in a saturator via a pump and an ejector. The air-saturated recirculation stream is then returned to the flotation unit through a throttling device. When the recirculated water mixes with the untreated wastewater in the flotation unit, the pressure drop across the throttling device causes the release of fine air bubbles, which attach to and float the poorly soluble metal compounds to the surface of the treated water in the form of flotation sludge.

The research involved an analysis of the theoretical relationships derived by the authors, taking into account the key parameters of the pressure flotation process, including the pressure in the saturator and the recirculation ratio, which is defined as the ratio of the recirculated water flow rate to the wastewater flow rate. These parameters determine the gas content in the flotation unit, as well as the concentration, size, and density of the floating particles.

## Conclusions

On the basis of a theoretical analysis of pressure flotation processes, the effectiveness of their application in the treatment of metal-containing water depends primarily on the ratio of the gas bubble concentration to the concentration of insoluble metal compounds (*G/S*). Our experimental results (Table 6) validated that the proposed system operates effectively at an optimized *G/S* ratio of 0.014.

To reduce energy costs by lowering the *G/S* ratio required for efficient flotation, it is necessary to apply molecular coagulation. During the flotation treatment of metal-containing water, conditions should be created that promote the formation of large, loosely structured flocs of sparingly soluble metal compounds. he study confirmed that the porous structure of these flocs, with densities close to water (1010–1030 kg/m³), ensures stable flotation even with minimal gas input.

Compared with the recirculation ratio, the air pressure in the saturator has a greater influence on flotation efficiency-due to the increase in gas content in the water-than does the recirculation ratio, making it a more economically advantageous option from an energy standpoint. For the tested wastewater, a pressure of 0.4 MPa and a recirculation ratio of 0.3 were identified as the most efficient parameters. In the case of flotation of smaller particles and at the same mass concentration of particles, an increase in the gas content in the treated water is needed.

A two-stage pressure flotation unit has been developed that enables the selective extraction of metals at different pH values corresponding to hydrate formation, with the possibility of their subsequent recycling from flotation sludge instead of disposal as environmentally hazardous metal-containing waste. At a technical scale of 15 m³/h, this enables the recovery of over 60 tonnes of metals annually.

The obtained theoretical relationships of the pressure flotation process make it possible to determine parameters that optimize the operating modes of flotation units, thereby ensuring the minimization of energy costs in the treatment of metal-containing water, in accordance with the requirements of the European Union best available techniques (BAT). Specifically, the two-chamber design demonstrated energy consumption 1.4–4.3 times lower than that of single-stage units.

Studies on the treatment of metal-containing water by pressure flotation have confirmed the high efficiency of this process, which is less energy intensive than the treatment of other categories of wastewater containing suspended solids.

## Data Availability

All the data included in this study are available from the corresponding author upon request.
